# Pathological complete response after intraperitoneal paclitaxel and systemic combined chemotherapy in a patient with peritoneal metastases from gastric cancer: a case report

**DOI:** 10.1186/s40792-020-00818-9

**Published:** 2020-03-30

**Authors:** Yoshiyuki Meguro, Hironori Yamaguchi, Joji Kitayama, Rihito Kanamaru, Shiro Matsumoto, Takashi Ui, Hidenori Haruta, Kentaro Kurashina, Shin Saito, Yoshinori Hosoya, Alan Kawarai Lefor, Naohiro Sata

**Affiliations:** 1 Department of Surgery, Division of Gastroenterological, General and Transplant Surgery, Jichi Medical University School of Medicine, Yakushiji 3311-1, Shimotsuke, Tochigi, 329-0498 Japan; 2 Department of Clinical Oncology, Jichi Medical University School of Medicine, Yakushiji 3311-1, Shimotsuke, Tochigi, 329-0498 Japan

**Keywords:** Gastric cancer, Peritoneal metastasis, Intraperitoneal chemotherapy, Paclitaxel, Pathological complete response

## Abstract

**Background:**

Despite recent progress in systemic chemotherapy, the prognosis of patients with peritoneal metastases from gastric cancer is still poor. Efficacious intraperitoneal and systemic combination chemotherapy regimens to treat patients with peritoneal metastases have recently been developed.

**Case presentation:**

A 74-year-old man with gastric cancer T4b (transverse mesocolon) N3 M1 (peritoneum) received combination chemotherapy with intraperitoneal administration of paclitaxel, intravenous oxaliplatin, and oral S-1. Eight courses of combined chemotherapy had remarkable anti-tumor effects on the primary lesion, lymph node metastases, and peritoneal metastases. Total gastrectomy with regional lymph node dissection was performed. Pathological examination revealed no viable tumor cells in the resected specimens. After gastrectomy, the patient received 25 courses of the same chemotherapy without oxaliplatin and has no evidence of recurrence 24 months later.

**Discussion:**

Therapeutic approaches including systemic chemotherapy, extended resection, and heated intraperitoneal chemotherapy have been used to treat patients with peritoneal metastases. Repeat therapy with intraperitoneal paclitaxel has been used recently. Intraperitoneal administration of paclitaxel results in prolonged retention in the peritoneal cavity with effects against peritoneal metastases. Repeated administration of paclitaxel does not cause adhesions in the peritoneal cavity. When combination chemotherapy is effective, salvage gastrectomy is a promising option with minimal morbidity and mortality.

**Conclusion:**

Combined chemotherapy with intraperitoneal paclitaxel and systemic chemotherapy followed by gastrectomy is a promising strategy for patients with advanced gastric cancer and peritoneal metastases.

## Background

Peritoneal metastases (PM) are a very poor prognostic factor in patients with advanced gastric cancer [1, 2]. Despite recent progress in systemic chemotherapy, the prognosis of patients with PM or positive peritoneal cytology is still poor. Recently, intraperitoneal and systemic combination chemotherapy regimens have been developed with efficacy against PM including ours [3]. We have also reported that surgery after a response to combination chemotherapy is a promising option for patients with PM or positive peritoneal cytology from gastric cancer [4, 5]. Here we present a patient with advanced gastric cancer and PM who achieved a pathological complete response using combined chemotherapy with intraperitoneal paclitaxel, intravenous oxaliplatin, and oral S-1.

## Case presentation

**Fig. 1:**
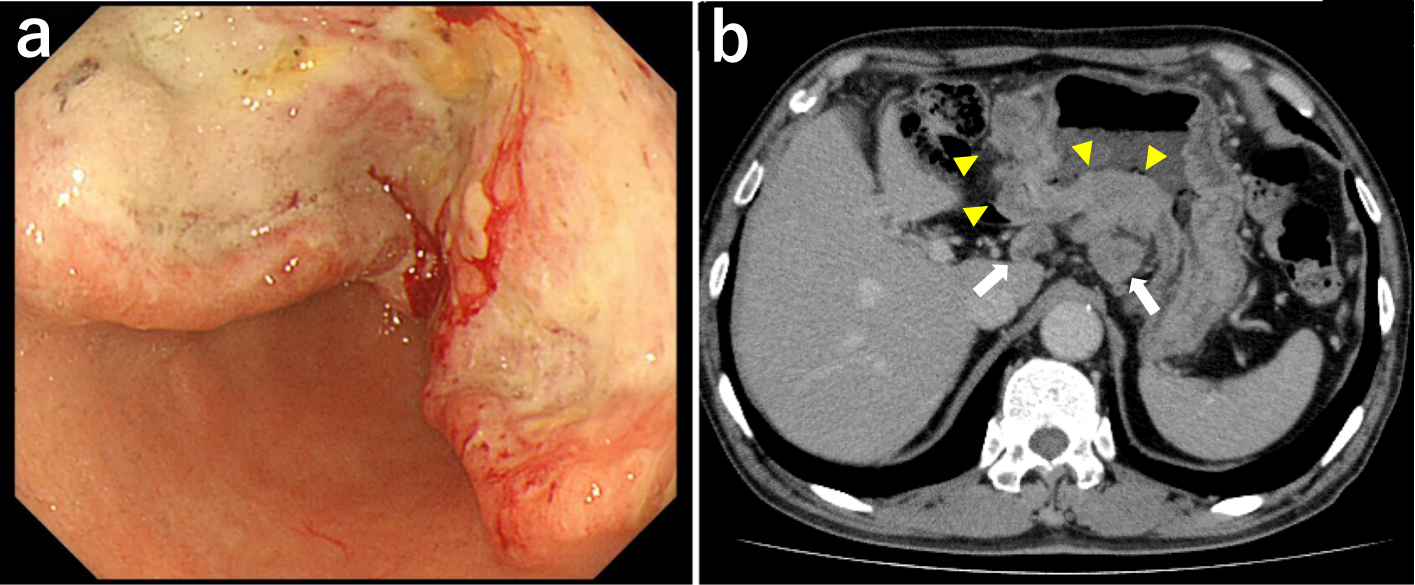
Imaging before combined therapy. **a** Gastroscopy showed a large Borrmann type II lesion in the middle and distal stomach. **b** Contrast-enhanced computed tomography scan showed thickening of the wall of the stomach (arrowhead) and enlarged regional lymph nodes (arrow)

**Fig. 2:**
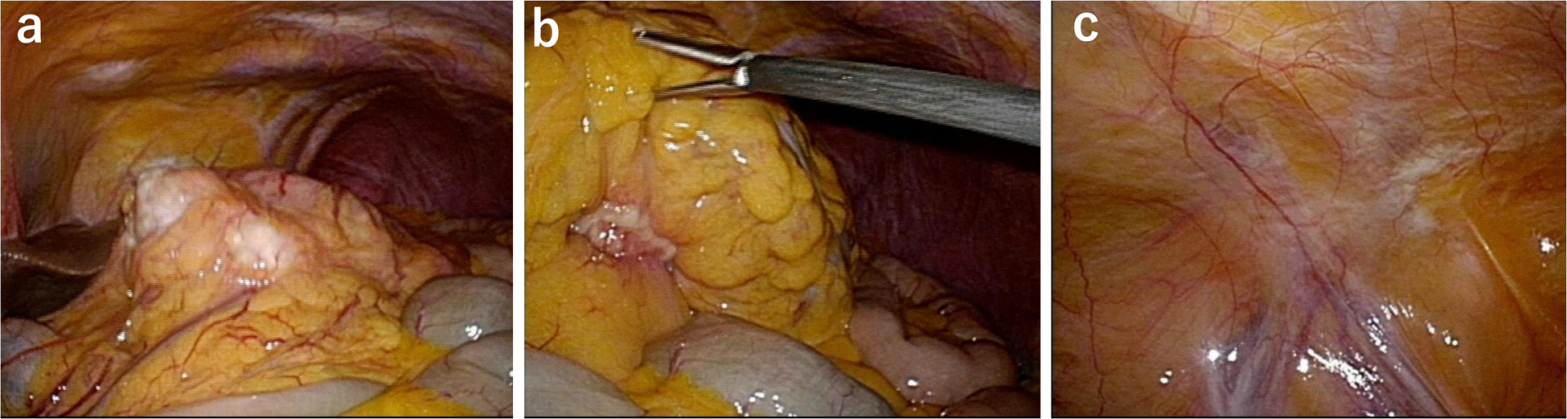
Laparoscopic images before combined therapy. **a** There are bulky regional lymph node metastases. **b** The transverse mesocolon is invaded by tumor. **c** Disseminated nodules are seen on the peritoneal surface

A 74-year-old man was referred for the treatment of gastric cancer. Gastroscopy showed a large Borrmann type II lesion in the middle and distal stomach (Fig. 1a). Biopsy revealed poorly differentiated adenocarcinoma with signet ring cell carcinoma. Contrast-enhanced computed tomography (CT) scan showed thickening of the wall of the stomach and enlarged lymph nodes consistent with lymph node metastases (Fig. 1b) without metastases to the liver or paraaortic lymph nodes. Staging laparoscopy showed regional lymph node metastases (Fig. 2a) and invasion of the transverse mesocolon (Fig. 2b). In addition, disseminated nodules were seen on the peritoneal surface in the abdominal cavity (Fig. 2c) with a peritoneal cancer index score of 7. Peritoneal fluid cytology was class III. The staging (UICC 8th edition) was T4b (transverse mesocolon) N3 M1 (peritoneum), stage IV. At the end of staging laparoscopy, an intraperitoneal access port was implanted in the subcutaneous space to allow for intraperitoneal chemotherapy administration. After staging laparoscopy, the patient received combined chemotherapy with intraperitoneal administration of paclitaxel, intravenous administration of oxaliplatin, and oral S-1. Oxaliplatin was administered intravenously at a dose of 100 mg/m^2^ on day 1, S-1 was administered orally twice daily at a dose of 80 mg/m^2^ per day for 14 consecutive days, followed by 7 days without treatment. On days 1 and 8, paclitaxel at a dose of 40 mg/m^2^ (maximum 60 mg) was diluted in 1 l of saline at room temperature and administrated intraperitoneally via the access port over 1 h. This chemotherapy regimen was approved as a clinical study by the Institutional Review Board of Jichi Medical University, and written informed consent was obtained from the patient for this therapy.

**Fig. 3:**
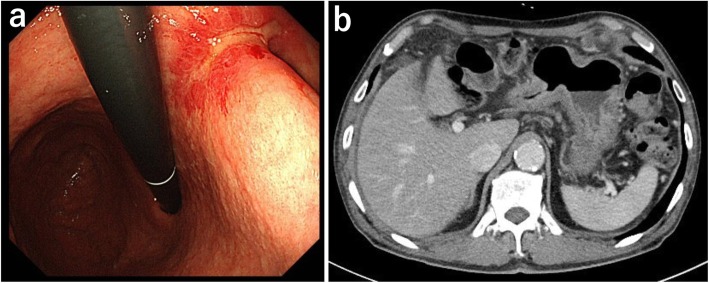
Imaging after combined therapy. **a** Gastroscopy showed only a small ulcer in the area of the original tumor. **b** Computed tomography scan showed reduced thickening of the gastric wall and marked shrinkage of regional lymph nodes

**Fig. 4:**
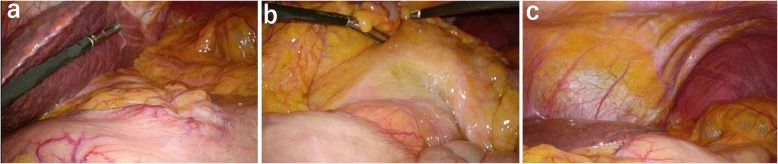
Laparoscopic images after combined therapy. **a** Disappearance of bulky regional lymph nodes metastases. **b** Invasion of the transverse mesocolon is not seen. **c** There are no disseminated nodules in the abdominal cavity

After eight courses of combined chemotherapy, gastroscopy showed only a small ulcer in the area of the original tumor (Fig. 3a), CT scan showed reduced gastric wall thickening, and regional lymph node metastases were markedly reduced in size (Fig. 3b). Distant metastases such as liver metastases or paraaortic lymph node metastases were not seen. A second laparoscopy showed that the regional lymph node metastases had resolved (Fig. 4a), invasion of the transverse mesocolon turned to scar (Fig. 4b), and peritoneal metastases were not macroscopically present (Fig. 4c). Cytological examination of peritoneal washings obtained during laparoscopy was negative for malignant cells. After a total of nine courses of combined chemotherapy, total gastrectomy with regional lymph node dissection was performed.

**Fig. 5:**
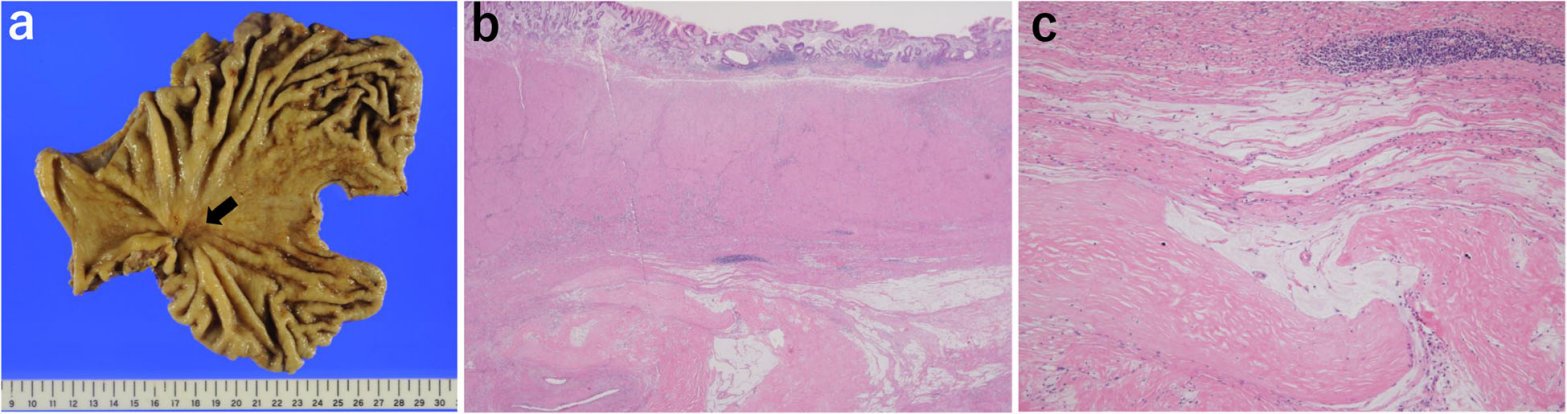
Gross and pathological findings of the resected stomach. **a** Gross findings of the resected stomach. There is a 35 × 30-mm ulcerated lesion in the middle and distal stomach (arrow). **b** Low-power view of the lesion. Histologically, there is a scar with fibrosis and hyalinization from the mucosal layer to the serosa (hematoxylin and eosin stain, original magnification × 20). **c** High-power view of the lesion. Some mucin-containing areas are seen; however, there are no viable cancer cells remaining in the primary lesion (hematoxylin and eosin stain, original magnification × 100)

Pathological examination of the specimen revealed no viable cancer cells remaining in the primary lesion (Fig. 5). Ten of 27 lymph nodes showed residual mucus, necrosis, and scar fibrosis, which are thought to represent a complete response to the chemotherapy. The postoperative course was uneventful. After gastrectomy, the patient received 25 courses of the same combined chemotherapy without oxaliplatin. He is alive at more than 24 months after surgery without evidence of recurrence.

## Discussion

PM from gastric cancer are difficult to treat, and the prognosis of patients with PM is still poor. According to the Japanese gastric cancer treatment guidelines 5th edition, 2018 [6], the combination of S-1 (tegafur, gimeracil, oteracil) or capecitabine with cisplatin or oxaliplatin is recommended for first-line chemotherapy, paclitaxel plus ramucirumab for second-line chemotherapy, then nivolumab or irinotecan for third-line chemotherapy for patients with PM. Recent progress in systemic chemotherapy has improved the prognosis of patients. However, the median survival time has been prolonged to only 1 year [5].

Probably because of the lack of measurable disease, there are few clinical trials specifically targeting patients with PM, and there is no established standard treatment [3]. So far, approaches such as systemic chemotherapy, extended resections, and heated intraperitoneal chemotherapy (HIPEC) have been conducted. The combination of cytoreductive surgery and HIPEC using drugs such as mitomycin, cisplatin, and oxaliplatin have been performed in specialized centers for many years. However, the median survival of patients is 9.2~11.5 months and the 1-year survival did not exceed 50%, except in a single small-scale phase II study [3]. The morbidity of these strategies was 14.7~88% with significant mortality, requiring strict patient selection [3].

In such a situation, attention has recently been drawn to repeated intraperitoneal chemotherapy using paclitaxel especially in Japan. Paclitaxel is insoluble in water and solubilized with a specific agent, Cremophor El® and ethanol. The size of paclitaxel particle in solution is relatively large (10–12 nm in diameter). With such features, intraabdominally administrated paclitaxel is not absorbed through vessels; however, it is absorbed slowly through the lymphatic system. This results in prolonged retention in the peritoneal cavity showing much higher area under the curve ratios than other hydrophilic drugs [3, 7–10]. Another merit of repeated intraperitoneal chemotherapy using paclitaxel is its characteristic of limiting adhesion formation. While cytoreductive surgery and HIPEC generally cause adhesions which prevent drugs from diffusing throughout the abdominal cavity, paclitaxel seldom causes peritoneal adhesions even if it is injected many times, presumably due to a strong antiproliferative effect on fibroblast cells [3]. Since it is considered that effects against the primary tumor and lymph node metastases are insufficient with only the intraperitoneal administration of paclitaxel, intraperitoneal and systemic combination chemotherapy regimens have been developed with efficacy against advanced gastric cancer with PM. With this approach, the median survival reached 15.1~24.6 months and the 1-year survival is over 70%, which is considerably better than the results with systemic chemotherapy or HIPEC [3]. Fujiwara reported favorable results with the regimen used in this patient in a phase II study of intraperitoneal paclitaxel plus S-1/oxaliplatin for gastric cancer with peritoneal metastasis (SOX+IP PTX trial). The 1-year OS rate was 71.5% (95% CI, 58–81%), the overall response rate was 67% (95% CI, 22–96%), and 75% of patients achieved disappearance of malignant cells on peritoneal cytology evaluation [11]. In addition, the results of a phase III trial comparing intraperitoneal and intravenous paclitaxel plus S-1 (IP arm) versus cisplatin plus S-1 (SP arm) in patients with gastric cancer with peritoneal metastasis (PHOENIX GC trial) were reported in 2018 [12]. This trial failed to show statistical superiority of intraperitoneal paclitaxel plus systemic chemotherapy possibly due to an unexpected imbalance that patients in the IP arm had significantly more ascites and the crossover from SP to IP. However, in an additional post hoc sensitivity analysis adjusted for baseline amount of ascites, overall survival was longer in the IP arm than in the SP arm (adjusted HR, 0.59; 95% CI, 0.39 to 0.87; *P* = .008). The 3-year overall survival rate was 21.9% (95% CI, 14.9 to 29.9%) in the IP arm and 6.0% (95% CI, 1.6 to 14.9%) in the SP arm. These results suggested possible clinical benefits of intraperitoneal PTX in patients with gastric cancer.

Grade 3/4 neutropenia occurred in 21~50% of patients undergoing repeated intraperitoneal chemotherapy [3]. According to one report, intraperitoneal access port-related complications occurred in 20.6%: inflow obstruction (7.6%), infection (6.9%), reflux (3.1%), subcutaneous masses (1.5%), and fistulae (1.5%). Generally, these were controllable and chemotherapy was not terminated by the development of these complications. In addition, survival was not affected by the presence or absence of port complications [13]. Considering these facts, repeated intraperitoneal chemotherapy is safe and can be tolerated with long-term sequential administrations, resulting in strong suppression against PM over a long period of time.

We reported that surgery after a response to combination chemotherapy is a promising option for patients with PM or positive peritoneal cytology from gastric cancer [4, 5]. Salvage gastrectomy was considered when a remarkable response to combination chemotherapy was observed in patients who would tolerate surgery. The indications for salvage gastrectomy include (1) negative peritoneal cytology, (2) disappearance or obvious shrinkage of peritoneal metastases, and (3) no unresectable metastasis identified by diagnostic imaging [5]. The timing of surgery is another important issue. On an empirical basis, we perform surgery on P0CY1 or P1, P2, and P3 patients (according to the Japanese classification of gastric carcinoma 12th edition) after 2–3, 6–8, and 9–18 courses respectively, with modification considering the timing of conversion of peritoneal cytology to negative, CT scan findings, and changes in the levels of serum tumor markers [5].

If gastrectomy is performed, the median survival reached 26.5~30.5 months with minimal morbidity and no mortality [4, 5]. This patient had the equivalent of P2 peritoneal metastases and received eight courses of combined chemotherapy. After that, gastroscopy, CT scan, and a second laparoscopy showed remarkable effect on the primary lesion and PM which fulfills the indications for surgery. In general, combination chemotherapy using the same regimen was restarted after the gastrectomy, as long as it remained effective with tolerable toxicity [4].

Achieving a pathologic complete response with chemotherapy alone in patients with peritoneal metastases from gastric cancer is a rare and valuable occurrence. So far, we have performed 15 salvage gastrectomies after combined chemotherapy at Jichi Medical University Hospital since 2016, but this was the only patient that achieved a pathologic complete response. In this case, it is reasonable to consider that surgery may not be necessary if a complete response is achieved, but we believe that tumor cells will survive even after this treatment in most case. Therefore, we believe it is necessary to perform cytoreductive surgery and adjuvant combined chemotherapy.

## Conclusion

We present a patient with advanced gastric cancer with PM, who achieved a pathologic complete response using combined chemotherapy with intraperitoneal paclitaxel, intravenous oxaliplatin, and oral S-1. Combined chemotherapy with intraperitoneal paclitaxel and systemic chemotherapy plus gastrectomy is a promising strategy that may be effective in patients with advanced gastric cancer with PM.

## Data Availability

Data sharing is not applicable to this article as no datasets were generated or analyzed during the current study.

## Abbreviations

PM: Peritoneal metastases
CT: Computed tomography
HIPE: Heated intraperitoneal chemotherapy
